# Automated diagnosis of 7 canine skin tumors using machine learning on H&E-stained whole slide images

**DOI:** 10.1177/03009858231189205

**Published:** 2023-07-29

**Authors:** Marco Fragoso-Garcia, Frauke Wilm, Christof A. Bertram, Sophie Merz, Anja Schmidt, Taryn Donovan, Andrea Fuchs-Baumgartinger, Alexander Bartel, Christian Marzahl, Laura Diehl, Chloe Puget, Andreas Maier, Marc Aubreville, Katharina Breininger, Robert Klopfleisch

**Affiliations:** 1 Freie Universität, Berlin, Germany; 2 Friedrich-Alexander-Universität Erlangen-Nürnberg, Erlangen, Germany; 3 University of Veterinary Medicine, Vienna, Austria; 4 IDEXX GmbH, Kornwestheim, Germany; 5 Schwarzman Animal Medical Center, New York, NY; 6 Technische Hochschule Ingolstadt, Ingolstadt, Germany

**Keywords:** computer-aided diagnosis, computational pathology, digital pathology, dog, machine learning, skin, veterinary oncology

## Abstract

Microscopic evaluation of hematoxylin and eosin-stained slides is still the diagnostic gold standard for a variety of diseases, including neoplasms. Nevertheless, intra- and interrater variability are well documented among pathologists. So far, computer assistance via automated image analysis has shown potential to support pathologists in improving accuracy and reproducibility of quantitative tasks. In this proof of principle study, we describe a machine-learning-based algorithm for the automated diagnosis of 7 of the most common canine skin tumors: trichoblastoma, squamous cell carcinoma, peripheral nerve sheath tumor, melanoma, histiocytoma, mast cell tumor, and plasmacytoma. We selected, digitized, and annotated 350 hematoxylin and eosin-stained slides (50 per tumor type) to create a database divided into training, *n* = 245 whole-slide images (WSIs), validation (*n* = 35 WSIs), and test sets (*n* = 70 WSIs). Full annotations included the 7 tumor classes and 6 normal skin structures. The data set was used to train a convolutional neural network (CNN) for the automatic segmentation of tumor and nontumor classes. Subsequently, the detected tumor regions were classified patch-wise into 1 of the 7 tumor classes. A majority of patches-approach led to a tumor classification accuracy of the network on the slide-level of 95% (133/140 WSIs), with a patch-level precision of 85%. The same 140 WSIs were provided to 6 experienced pathologists for diagnosis, who achieved a similar slide-level accuracy of 98% (137/140 correct majority votes). Our results highlight the feasibility of artificial intelligence-based methods as a support tool in diagnostic oncologic pathology with future applications in other species and tumor types.

The use and interpretation of hematoxylin and eosin (HE)-stained slides form the basis of diagnostic pathology since the 19th century.^28^ HE-based histologic diagnosis is also still the gold standard diagnostic tool for tumor diagnosis and subtyping, as well as for surgical margin evaluation and tumor grading.^19^ Despite its gold standard designation, its inherent intra- and interobserver variability is a common and well-known shortcoming of the pathologist for both qualitative diagnosis (e.g. tumor subtyping) and quantitative tasks (e.g. mitotic count for tumor grading).^4,6^

Computer-aided diagnosis (CAD) and automatic image analysis using software solutions can be considered complementary to the pathologist workflow. The addition of these technologies so far is most helpful for improving the quality of repetitive quantitative tasks in histopathology and cytology.^1,4,5,20,26,30^ With the availability of advanced deep learning methods, it is now possible to train algorithms that can identify more complex structures such as primary tumors and metastases in breast cancer (human pathology),^11,27^ detect mitotic figures (both human and veterinary pathology),^5,23^ classify round cell tumors (veterinary pathology),^25^ classify and identify of melanocytic lesions (human pathology),^21^ and quantify intracellular pigment in cytological slides (veterinary pathology).^7,17,18^

This study aimed at taking a first step toward creating an algorithm to automatically detect and classify common canine skin tumors.^13,16,19^ We hypothesize that the training of an artificial neural network using an appropriate number of well-annotated digital images of canine cutaneous tumors will lead to a software solution that identifies and differentiates common canine cutaneous tumor types with a similar sensitivity and specificity as trained pathologists.

## Material and Methods

### Case Selection and Scanning

Surgical biopsies of 7 of the most frequent tumors in dogs were retrospectively selected from the histopathology archive of the Institute of Veterinary Pathology of the Freie Universität Berlin. The tumor types were trichoblastoma, squamous cell carcinoma (SCC), melanoma, peripheral nerve sheath tumor (PNST), mast cell tumor (MCT), plasmacytoma, and histiocytoma. Seventy cases per tumor were chosen with respect to typical histological features, acceptable state of preservation, sufficient histological perceptibility of cellular details, and staining quality (HE, total *n* = 490 slides). Melanoma slides were 45% heavily pigmented melanomas, 35% with less than 50% pigment, and 20% amelanotic melanomas. In addition, 18/70 (26%) melanomas were of dominant spindloid morphology, 40/70 (57%) melanomas of dominant epithelioid morphology, 4/70 (6%) of dominant balloon cell morphology, and 8/70 (11%) with similar contribution of spindle and epithelioid cells.

All glass slides were digitized as whole slide images (WSI) using a linear scanner (ScanScope CS2, Leica) in 1 focal plane with default settings at a magnification of 400× (image resolution: 0.25 μm/pixel). WSI were viewed using ImageScope (Leica) during all phases of the project.

### Data Sets

350 slides (50 of each tumor type) were randomly attributed to data set 1, which was used for training (*n* = 245 WSIs), validation (*n* = 35 WSIs), and testing (*n* = 70 WSIs) of the convolutional neural network (CNN, method described below).^29^ The remaining 140 slides (20 slides per tumor type, data set 2) were used as a test set to compare the algorithm and the pathologists’ performance regarding a slide-level assessment of tumor type.

### Annotations of Tissue Area and Tumors

Each slide of data set 1 was fully annotated using 6 classes of nonneoplastic structures (epidermis, dermis, subcutis, inflammation-necrosis, bone, and cartilage) and 7 classes of neoplastic structures (trichoblastoma, SCC, melanoma, PNST, MCT, plasmacytoma, and histiocytoma) (Supplemental Table S1) by 3 independent annotators.^29^ Annotations were performed in SlideRunner,^3^ a software for annotations on WSIs. Using the polygon tool, each area of interest was surrounded with a thin line, from point to point until it was completely delimited.

In total, 12,424 annotations were made, with an annotation area of 76,118.05 mm^2^ (Supplemental Table S1).

### Development of the Algorithm: Training and Testing

Technical method development was conducted at the Pattern Recognition Lab at the Friedrich-Alexander-Universität Erlangen-Nürnberg in close collaboration with the medical experts at the Freie Universität Berlin.^29^

A semantic segmentation algorithm was trained to distinguish between background, nontumoral skin structures (epidermis, dermis, subcutis, and inflammation combined with necrosis), and tumor (regardless of tumor type). For segmentation, a UNet^22^ architecture with a ResNet18^14^ backbone pretrained on ImageNet^24^ was used. The network was trained with image patches sized 512 × 512 pixels and a resolution of 4.0 μm/pixel. For additional implementation details we refer to the work of Wilm et al.^20^ Due to high quantitative (area) class-imbalances, an adaptive sampling strategy was used. Initially, 10 patches per slide were sampled uniformly across all annotation classes, resulting in 2,450 training patches. These were used to train the network for 1 epoch. Then, the network performance was evaluated on 350 validation patches (10 per WSI) sampled in the same fashion. Afterwards, the probability of sampling patches from a class with a low validation performance was increased, whilst high-performing classes were under-sampled. Using this adaptive sampling scheme, the model was explicitly trained on classes facing most difficulties, aiming for faster convergence of the model training. The model was trained for 100 epochs with a maximal learning rate of 10-4 and a batch size of 4. As loss function, a combination of cross-entropy and Dice loss was used.

In a second step, a tumor type classification network was trained to distinguish between the 7 tumor types. The same data set split as used for training the segmentation network was used, resulting in 35 training WSIs per tumor type. Due to the high morphological resemblance of round-cell tumors, which might only be distinguishable at a high image resolution, the classification network was trained on patches at the original resolution of 0.25 μm/pixel. To cover as much context as possible, the input size of the model was increased to 1024 × 1024 pixels. An EfficientNet-B5^31^ based classification architecture pretrained on ImageNet^24^ was used. For each epoch, 10 patches per slide were sampled, ensuring that each tumor was represented equally and avoiding class-imbalances across all tumor types. The network was additionally trained to predict a “nonneoplastic” class, for which patches from all remaining annotation classes (epidermis, dermis, subcutis, and inflammation combined with necrosis) were used. A patch was only used for training the classification network if at least 90% of the pixels were annotated as the sampled class. A batch size of 4 and a maximal learning rate of 10^3^ were used to train the network for 100 epochs which ensured convergence of the training. For optimization, the cross-entropy loss and the Adam optimizer were used.

**Figure 1.**
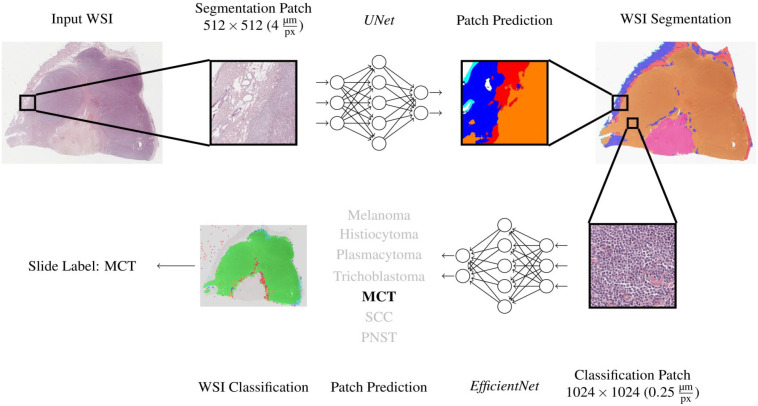
Pipeline for the tissue segmentation and tumor classification algorithms. For training of the algorithm, whole slide images (WSI, tumor [all classes] and nontumor areas) of the training data set were cut into smaller patches and provided to a first artificial neuronal network (UNet), which was developed to segment tumor from nontumor areas for reduction of WSI complexity. Subsequently, segments classified as “tumor” were divided into the 7 tumor classes to train a second neuronal network (EfficientNet) for tumor classification. Both algorithms were finally tested on a validation set of 20 tumors. MCT, mast cell tumor; SCC, squamous cell carcinoma; PNST, peripheral nerve sheath tumor.

Fig. 1 visualizes the WSI inference pipeline. A slide was first segmented into 6 classes using the segmentation network. Afterwards, regions segmented as tumors were classified into 1 of the 7 tumor types. For this, the predicted tumor region was upscaled from the segmentation resolution of 4 μm/pixel to the classification resolution of 0.25 μm/pixel. Then, the tumor region was divided into patches sized 1024 × 1024 pixels, which were only passed on to the classification network if they were completely segmented as tumor. Each patch then obtained a classification label by the network. All patches classified as nonneoplastic tissue were excluded, while all remaining patch classifications were combined to a slide classification label using majority voting.

In the same way, the algorithm was run on the second data set, which did not contain pixel-level annotations, only hidden diagnostic labels for each WSI.

### Pathologist Consensus Diagnoses

Six experienced pathologists (different from the ground truth pathologists) were asked to provide a primary diagnosis and 2 differential diagnoses for the second data set, which contains 140 WSIs (20 per tumor type). For each of the 3 diagnoses, the pathologists had to provide a value of the experienced certainty/confidence level (possible range 0.01–0.98, sum of the 3 values had to be 1.0, primary diagnosis had to be associated with the highest confidence level). The diagnoses were subsequently ranked by the confidence level value in the following order: primary diagnosis, first differential diagnosis, and second differential diagnosis.

For the consensus diagnosis of the pathologist group, a simple majority vote was calculated. The tumor type with the highest number of votes was defined as primary consensus diagnosis. First and second differential diagnoses were defined accordingly. In the case of a draw, the tumor type with the highest average confidence level was selected. A tumor was thus able to receive a maximum of 20/20 correct diagnoses (according to the ground truth) in 1 tumor group.

To display the variation in the strength of the consensus for the different tumor types, an accumulated decimal consensus level (decimal consensus) per tumor and accumulated per tumor group was calculated. For example, if 4/6 pathologists chose the final consensus diagnosis and 2/6 chose another tumor type on slide 1 of the respective tumor group, the decimal consensus resulted in 4.0 for this tumor (range 0.0–6.0). If for slide 2 of this tumor group 5/6 pathologists chose the final primary diagnosis and 1/6 pathologists chose a different diagnosis, the decimal consensus for this slide would be 5.0 and the accumulated consensus level for the overall tumor type would be 4.5, sum of (4 + 5) / 2.

### Statistical Analysis

All statistical analyses were performed using SPSS (v28) or customized software solutions programmed by A.B. Slide-level recall (sensitivity) was defined as the sum of true positive consensus or algorithmic diagnosis over the sum of true-positive and false-negatives diagnoses (consensus or algorithm) in all slides of 1 tumor type. For patch-level recall, the percentage of correctly classified patches (true positive) by the algorithm over the sum of percentage of true-positively and false-negatively diagnosed patches in all cases of 1 tumor type was used.

Slide-level precision (positive predictive value) was defined as the sum of true positive diagnoses (consensus or algorithm) over the sum of true- and false-positive diagnoses. Patch-level precision was defined as the percentage of correctly algorithmically classified patches over the sum of true-positively and false-negatively diagnosed patches in all cases of 1 tumor type.

F1 score (a measure of classification accuracy) is defined as the harmonic mean of precision and recall considering false-negatives and false-positives for algorithmic and pathologists’ diagnoses. For algorithmic diagnosis only the first 3 algorithm classifications with the highest number of associated patches in the respective were considered.

The average confidence level of the pathologists (range 0.01–0.98 per tumor, total 1.0) of the 3 diagnoses of the pathologists and the percentage of the 3 top patch diagnoses of the algorithm, respectively, per tumor were used to perform a principal component analysis (PCA).^24,25,31^ Two separate analyses were performed, one for the pathologists and one for the algorithm. Biplots were generated to show how similar pathologists appreciated single tumors within the tumor classes (small/dense clusters meaning high similarity) and between the 7 tumor classes (close or overlapping clusters meaning high similarity of 2 tumor clusters). For the algorithm biplot, small/dense clusters reflect a similar distribution of patch diagnoses in the 20 cases of 1 class, while close or overlapping clusters reflect overlapping similar single patch diagnosis distributions between tumor classes. Biplots were created using the package ggbiplot (version 0.55) in R version 4.1.3 (R foundation Vienna).

For the visualization and evaluation of the model performance and its comparison with the pathologists’ responses, confusion matrices were created. Confusion matrix (CM) on the slide-level diagnosis for algorithmic and pathologist’s diagnosis and on patch-level diagnosis for algorithmic diagnosis were calculated.

### Immunohistochemistry

To confirm the cases in our database and determine their individual ground truth, a conventional immunohistochemistry analysis (IHC) analysis was performed for selected cases. The following antibodies were used: Melan-A (Dianova, A103) monoclonal mouse for melanoma^10^ in a 1:300 dilution. CD79acy monoclonal mouse anti-human for plasmacytoma (Dako, HM57) in a 1:60 dilution. CK10 (Abcam, EP1607IHCY) monoclonal rabbit for SCC in a 1:1000 dilution. E-Cadherin (Abcam, EP913[2]y) monoclonal rabbit for histiocytoma in a 1:1000 dilution. Briefly, all slides were pretreated using citrate buffer and microwave and signals were visualized using the 3, 3’-diaminobenzidine (DAB) method. As negative control, slides were treated with albumin containing distilled water instead of the primary antibodies. Archive morphologically prototypical tumor was used as positive controls. In the case of trichoblastoma and PNST, IHC was not necessary. In the case of MCT, IHC was not performed because the diagnosis was made by exclusion by comparison with the markers for round cell tumors (plasmacytoma and histiocytoma).

## Results

### Algorithmic Tissue Segmentation

The test split (*n* = 10 WSIs per tumor type, total 70 cases) of the fully annotated data set 1 was used to evaluate the algorithm performance in segmenting tumor and nontumor classes.

Algorithmic segmentation of tumor from all other tissue classes had the highest precision of 95% but the lowest recall of 66% (F1 score 0.78, Supplemental Table 2). Thus, nontumor areas were more often diagnosed as tumor than tumor areas as nontumor areas. Subcutis was segmented with an 85% precision (F1 score 0.85), followed by dermis (84%, F1 score 0.76) and epidermis (79%, F1 score 0.87). The class with the lowest precision was inflammation/necrosis (I/N) with a 46% precision (F1 score 0.62). The confusion of this class was mainly with tumor class (false-positive tumor diagnosis in 26% of all I/N patches. I/N also had overlaps with dermis (15%) and subcutis (11%), but rarely with epidermis (2%).

### Tumor Classification—Algorithmic Versus Human Consensus Diagnosis

**Figure 2.**
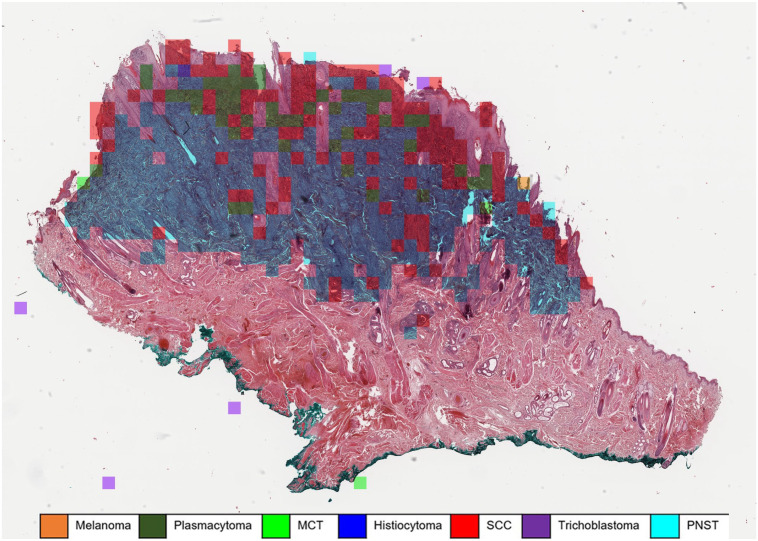
Automatic classification of a histiocytoma using a machine learning algorithm. The majority of patches are correctly classified as histiocytoma (blue overlay). However, approximately 22% of the tumor patches were classified as squamous cell carcinoma (SCC) (red overlay), which are mostly superficially located fragments of hyperplastic epidermis. Whole slide image, hematoxylin and eosin. MCT, mast cell tumor; PNST, peripheral nerve sheath tumor.

Automated algorithmic tumor classification was performed on the nonannotated data set 2 (*n* = 140 WSIs, 20 per tumor type) and later compared with the pathologist consensus. The algorithm pipeline depicted in Fig. 1 resulted in multiple patch predictions per WSI (Fig. 2). To convert the patch-wise classifications into a main diagnosis and 2 differential diagnoses per tumor case, a simple ranking of the absolute patch number associated with the respective tumor class was performed. The tumor class with the highest number of associated patches in 1 WSI was defined as the primary diagnosis. The differential diagnoses 1 and 2 were identified accordingly. The remaining diagnoses on patch level were usually distributed over more than 3 types of tumors, but with low percentages.

A diagnosis on the slide-level was a majority vote by the pathologists or by the class with the highest number of algorithmic assigned patches. With this approach the performance of the pathologist group and the algorithm were compared on data set 2 (20 WSI per tumor type).

**Figure 3.**
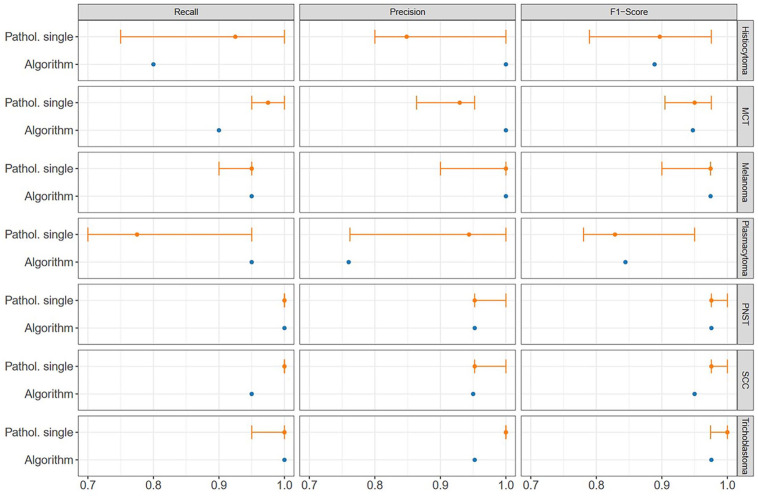
Overview of recall (sensitivity), precision (positive predictive value), and F1 score of the individual diagnoses of the pathologists and algorithm by tumor type. The median of the pathologists is shown as a point; the error bars show the range between all pathologists, from the minimum (0.7) to the maximum scored (1.0). MCT, mast cell tumor; PNST, peripheral nerve sheath tumor; SCC, squamous cell carcinoma.

The algorithm correctly classified 133/140 cases (95%). The consensus diagnosis of the pathologists classified 137/140 cases (98%) correctly with a total decimal consensus of 5.8/6 correct answers. Comparison of the median F1 score for trichoblastoma (pathologists: 1.00, algorithmic: 0.98), SCC (pathologists: 1.99, algorithmic: 0.98), PNST (pathologists: 0.98, algorithmic: 0.98), melanoma (pathologists: 0.97, algorithmic: 0.97), and mast cell tumor (pathologists: 0.95, algorithmic: 0.95) diagnosis by pathologists’ consensus and algorithmic diagnosis found similar high scores (Fig. 3, Table 1). Two of the round cell tumors, histiocytoma (pathologists: 0.89, algorithmic: 0.89) and plasmacytoma (pathologists: 0.83, algorithmic: 0.84), were tumors with the lowest F1 score for both pathologists and the algorithm.

**Table 1.** Comparison of the combination of recall and precision (F1 score) with respect to the pathologists’ consensus and the algorithm classification.

Tumor Type	F1 Score Median of 6 Pathologists’ Median (Range)	F1 Score Algorithm
Histiocytoma	0.90 (0.789–0.976)	0.89
MCT	0.95 (0.905–0.976)	0.95
Melanoma	0.97 (0.900–0.974)	0.97
Plasmacytoma	0.83 (0.780–0.950)	0.84
PNST	0.98 (0.976–1.000)	0.98
SCC	0.98 (0.976–1.000)	0.95
Trichoblastoma	1.00 (0.974–1.000)	0.98

Abbreviations: MCT, mast cell tumor; PNST, peripheral nerve sheath tumor; SCC, squamous cell carcinoma.

**Figure 4.**
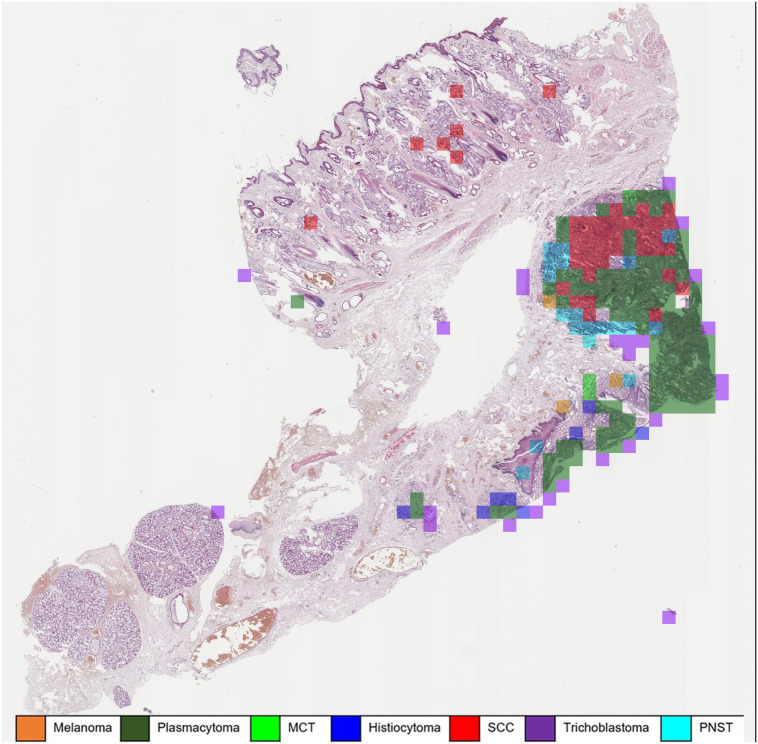
Automatic classification of a squamous cell carcinoma (SCC). Highly inflamed areas in the SCC (red) are (mis)interpreted as plasmacytoma (green) or occasionally histiocytoma (blue) due to severe chronic inflammation of the tumor. Whole slide image, hematoxylin and eosin. MCT, mast cell tumor; PNST, peripheral nerve sheath tumor.

Confusion matrix analysis (slide-level diagnosis, Table 2) showed that pathologists misinterpreted 2 melanoma cases as PNST or SCC, respectively, and 1 plasmacytoma as MCT (Table 2B). Similarly, the algorithmic diagnosis misinterpreted 1 melanoma as PNST. Two SCC were misinterpreted as plasmacytoma, mainly because of the strong inflammation in these tumors (Fig. 4). Two plasmacytomas were misinterpreted as melanoma or SCC and thus not as expected as a round cell tumor differential diagnosis like histiocytoma or MCT (Table 2A).

**Table 2.** Confusion matrix on the slide level of the algorithm (A) and the pathologists (B) against the ground truth (number of cases with the respective diagnosis).

A—Algorithm							
Algorithm Versus Ground Truth	Melanoma	Plasmacytoma	MCT	PNST	SCC	Trichoblastoma	Histiocytoma
Melanoma	19	0	0	1	0	0	0
Plasmacytoma	1	18	0	0	1	0	0
MCT	0	0	19	0	1	0	0
PNST|	0	0	0	20	0	0	0
SCC	0	2	0	0	18	0	0
Trichoblastoma	0	0	0	0	0	20	0
Histiocytoma	0	1	0	0	0	0	19
B—Pathologist’s Consensus							
Pathologists Ground Truth	Melanoma	Plasmacytoma	MCT	PNST	SCC	Trichoblastoma	Histiocytoma
Melanoma	18	0	0	1	1	0	0
Plasmacytoma	0	19	1	0	0	0	0
MCT	0	0	20	0	0	0	0
PNST	0	0	0	20	0	0	0
SCC	0	0	0	0	20	0	0
Trichoblastoma	0	0	0	0	0	20	0
Histiocytoma	0	0	0	0	0	0	20

Abbreviations: MCT, mast cell tumor; PNST, peripheral nerve sheath tumor; SCC, squamous cell carcinoma.

Confusion matrix analysis on the patch-level (percentage of correctly/incorrectly classified patches, Table 3) showed that between 70% (SCC) and 95% (MCT) of the tumor patches (average over all tumor classes 85%) were correctly classified. The falsely classified patches were rather evenly distributed over the other, wrong, tumor classes. Exceptions with an accumulation of wrong classifications in 1 class were 12% of plasmacytoma patches classified as histiocytoma, 9% of histiocytoma patches classified as MCT and 9% of SCC patches classified as MCT areas.

**Table 3.** Confusion matrix of the algorithm’s predictions at the patch level (percentage of patches with the correct diagnosis).

Algorithm Versus Ground Truth	Melanoma	Plasmacytoma	MCT	PNST	SCC	Trichoblastoma	Histiocytoma
Melanoma	0.91	0.02	0.00	0.02	0.04	0.02	0.00
Plasmacytoma	0.05	0.75	0.01	0.01	0.06	0.01	0.05
MCT	0.02	0.03	0.95	0.02	0.09	0.00	0.09
PNST	0.00	0.04	0.01	0.91	0.07	0.00	0.02
SCC	0.02	0.03	0.00	0.03	0.70	0.02	0.03
Trichoblastoma	0.00	0.02	0.00	0.01	0.01	0.94	0.01
Histiocytoma	0.00	0.12	0.00	0.01	0.03	0.00	0.80
Precision	0.91	0.75	0.95	0.91	0.70	0.94	0.80
Recall	0.90	0.79	0.80	0.86	0.85	0.94	0.83
F1 score	0.91	0.77	0.87	0.88	0.77	0.94	0.81

Values < 0.01 are presented as 0.00.

Abbreviations: MCT, mast cell tumor; PNST, peripheral nerve sheath tumor; SCC, squamous cell carcinoma.

**Figure 5.**
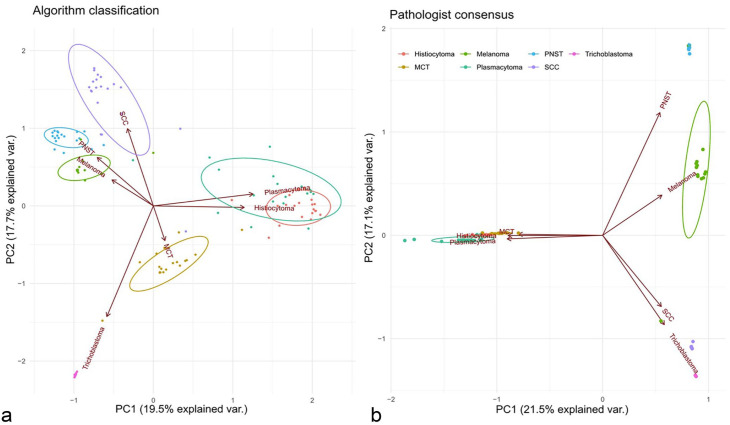
Biplot of the principal component analysis of the top 3 diagnoses for all tumors. (a) Distribution of algorithm classification regarding the tumor type based on the patch-level diagnosis percentages. (b) Distribution of pathologists’ consensus based on their combined differential diagnosis certainty estimates. Each diagnosis (classification regarding the algorithm and diagnosis consensus regarding the pathologists) is visualized as a point, with color denoting its tumor type (*n* = 140 whole slide images). The denser the points are arranged (delineated areas) the higher the appreciated similarity of the tumors. In addition, clusters close to each other or overlapping indicate a close appreciated similarity between the 2 tumor classes. PNST, peripheral nerve sheath tumor; MCT, mast cell tumor; SCC, squamous cell carcinoma.

PCA of the 3 differential consensus diagnoses by the pathologists and their associated average confidence level found invariably high confidence levels of the pathologist for their tumor diagnosis (dense/small clusters) (Fig. 5). As expected, the round cell tumors plasmacytoma, histiocytoma and MCT were appreciated as similar to each other (clusters close to each other or overlapping) but very dissimilar from the other tumors (distant from the other clusters and long vector distance). Similarly expected, the epithelial tumors SCC and trichoblastoma were appreciated as similar to each other but very dissimilar from the other tumors. The pathologists were thus mostly very confident in their diagnosis and found round cell tumors as most similar.

PCA of the 3 algorithmic diagnoses using the ranked 3 tumor classes with the highest patch number and the portion of different patch classes in each slide found some similar but also different results compared with the human consensus (Fig. 5). In agreement with the human consensus, the 2 round cell tumor clusters plasmacytoma and histiocytoma were overlapping, thus appreciated as similar (Fig. 5). However, the third class of round cell tumors, MCT, was identified as clearly different from the other 2 round cell classes. Also, in contrast to the human diagnosis, trichoblastoma was recognized as not highly similar to the other epithelial tumor SCC. In general, for algorithmic diagnoses the clusters areas were larger (less similarity = less dominance of 1 tumor class patch within the tumor area) and vectors were shorter (higher similarity between the tumor classes). Thus, although the algorithm mostly came to the same slide level diagnosis, the basis of this diagnosis (percentage of correct positive patches) was more diverse than the confidence levels of the pathologists.

However, comparison of pathologist confidence level in the primary correct diagnosis with the portion of algorithmically correctly diagnosed patches (according to the ground truth) found a similar variation for the different tumor classes, except for SCC (Table 4). Melanoma, PNST, trichoblastoma, and MCT showed both high confidence levels (≅0.9) and a high portion (⪆0.9) of correctly classified patches.

**Table 4.** Comparison of pathologist confidence level in correct diagnosis with portion of algorithmically correctly diagnosis patches.

Tumor Class	Average Confidence (0–1) of 6 Pathologists in Their Diagnosis	Portion (0–1) of Correctly Positive Patches Per Tumor Subtype
Melanoma	0.86	0.91
PNST	0.96	0.91
SCC	0.95	0.70
Trichoblastoma	0.97	0.94
Plasmacytoma	0.82	0.75
MCT	0.93	0.95
Histiocytoma	0.87	0.80

Abbreviations: PNST, peripheral nerve sheath tumor; SCC, squamous cell carcinoma; MCT, mast cell tumor.

## Discussion

This study aimed to develop of a machine-learning based algorithm that is able to diagnose the most common canine skin with a similar accuracy as experienced veterinary pathologists. Challenging 6 pathologists and the algorithm with 140 cases of 7 different tumor classes confirmed that the developed algorithm diagnosed 95% of the cases correctly, which was only 3% less accurate than 98% correct consensus (majority votes) diagnoses by the pathologists. The algorithm, therefore, showed a mildly lower accuracy but was still successful on a very high level.^6^

Comparison of the algorithmic diagnoses with the human diagnoses is not straightforward. Both are based on the ranking of the 3 dominant diagnoses, but also on the subjective confidence level (in the case of humans) with a winner-takes-all approach, whereas in the case of the algorithm, it is based on a majority vote over the percentage of patches identified on the slides. However, there are some correlations between the human consensus and algorithmic diagnosis, which may give hints on the way the algorithm but also the human observer comes to a diagnosis.

Assuming that a lower portion of patches of the primary/correct tumor class indicates “uncertainty/low confidence” of the algorithm, a rather artificial but interesting comparison of the human with the algorithm is possible (besides the pure percentage of correct whole slide level diagnoses).

In general, a major difference between the human and the algorithm, was a less “confident” diagnosis by the algorithm as is indicated principal component analysis (Fig. 5). Furthermore, while all pathologists agree that epithelial tumors (/round cell tumors) look more similar to each other than to round cell tumors (/epithelial tumors), algorithmic analysis did not completely come to the same conclusion. While the round cell tumors plasmacytoma and histiocytoma were appreciated as similar, the round cell tumor type “mast cell tumor” was appreciated as more similar to trichoblastoma than to the other round cells by the algorithm, which is not fully understandable from a human point of view. Also, the epithelial tumor SCC was found more similar to the spindle cell tumor PNST and the neuroectodermal tumor melanoma than to the other epithelial tumor, trichoblastoma, by the algorithm. This could be explained by the histological complexity of SCC of tumor, with the presence of inflammation and desmoplasia, in different degrees of differentiation.^8^

There may be 2 reasons for this discrepancy between the digital and the human diagnostician. First, cognitively/psychologically, humans may overestimate their confidence levels once they decide for a primary diagnosis and thus forget potential doubts on the way to the diagnosis.^6,12^ In contrast, the patch distribution found by the algorithm is objective^15^ and not overwritten postdiagnosis by a final subjective confidence level. Most probably, the human observer will also find large areas in typical tumors that are not typical and diagnostic (e.g. inflammation, necrosis, desmoplasia, and fibrosis). However, by conscious decision or due to a cognitive “tunnel vision/anchoring” effect, the human seems to ignore these areas and only focus on the areas relevant for the final diagnosis.^2^ This hypothesis is currently being investigated by our group in an independent study.

Second, the algorithm seems to base diagnostic decisions for epithelial or round cell tumors on other morphologic features than the human. The general roundish or polygonal or spindloid cell shape may be less important for digital algorithmic decisions than other, so far unknown, features (nuclear shape, staining intensity, morphology of the stroma). Methods of explainable AI, indicating areas with relevant features for the algorithm, may help to identify and translate these features for human cognition.^9^

Nevertheless, as shown in Table 3, tumor types with a lower portion of digitally diagnosed correct patches associated with the ground truth tumor class were also associated with a lower confidence in the primary decision by the human observer. This could indicate that there may be parallels in the decision process of the human mind similar to those of the algorithmic patch-wise decision.

The only exception from this generalization was the SCC class. Here, the algorithm had lower SCC patch portions (0.7) than the confidence of the pathologists (0.95). Thus, 30% of the tumor area was classified into other classes, mainly plasmacytoma, PNST, and MCT. Given the frequently observed inflammation, necrosis and the tumor stroma in SCC, the 30% of patches may thus not be misdiagnosed but indicate the inhomogeneity of SCC.^19^

This exemplifies that in our 2-step segmentation-classification approach, simple majority decision for tumor subtypes relies on a very precise preselection of tumor areas. Otherwise, automatic tumor classification, which is built to force each patch presented into 1 of the 7 tumor classes independent from its true nature, may lead to a wrong diagnosis. For instance, strong inflammation in an epithelial or spindle cell tumor could lead to a misclassification and confusion with any of the round cell tumors. In a follow-up project we are developing a broad data set of possible inflammatory lesions in canine skin to increase the segmentation accuracy of inflammation versus tumor and to further classify different inflammation types in the skin.

Alternatively, a 1-step approach containing all potential diagnoses in a biopsy may prevent the consequential error of imperfect segmentation on classification. However, this requires very large training data sets that contain either all possible histomorphological skin diagnoses, which will prove difficult given the complexity of dermatologic pathology or it has to identify at least all potential morphologic features (all possible inflammatory cells, all forms of necrosis, degeneration, etc.). In this case, automatic diagnosis may be presented in (quantitative) patterns of features present that may be specific for a certain inflammatory, degenerative, or neoplastic disease. The resulting set of identified features then must be evaluated and weighed by a responsible pathologist or automatically according to the clinical data/questions and the prognostic/therapeutic relevance of the different features. Weighing of features/subdiagnoses will be highly relevant, to avoid, for instance, missing a small area of auricular SCC in a large area of highly inflamed auricular skin.

In conclusion, this study prototypically shows that a machine-learning-based algorithm is able to segment tumor from nontumor areas and subsequently classifies a small subset of canine skin tumors with similar, although minimally lower, accuracy compared with the consensus of 6 experienced pathologists. Statistical analysis found differences in the appreciated similarity of tumor types by the algorithm and the pathologists, which points toward different relevant morphologic features for diagnosis used by the algorithm. However, the percentage of patches correctly algorithmically classified in a tumor correlated coarsely with the subjective confidence level of confidence of the pathologists (for a tumor), indicating potentially similar “cognitive” processes in both diagnostic approaches. A majority-vote for patch number-based algorithmic decisions for final diagnosis proved successful in the current approach but the decision process may have to be refined if automatic diagnosis is challenged with a larger set of potential diagnoses and less prototypical biopsies.

## Supplemental Material

sj-pdf-1-vet-10.1177_03009858231189205 – Supplemental material for Automated diagnosis of 7 canine skin tumors using machine learning on H&E-stained whole slide images Click here for additional data file. Supplemental material, sj-pdf-1-vet-10.1177_03009858231189205 for Automated diagnosis of 7 canine skin tumors using machine learning on H&E-stained whole slide images by Marco Fragoso-Garcia, Frauke Wilm, Christof A. Bertram, Sophie Merz, Anja Schmidt, Taryn Donovan, Andrea Fuchs-Baumgartinger, Alexander Bartel, Christian Marzahl, Laura Diehl, Chloe Puget, Andreas Maier, Marc Aubreville, Katharina Breininger and Robert Klopfleisch in Veterinary Pathology
