# Supplementary material for: Automated diagnosis of 7 canine skin tumors using machine learning on H&E-stained whole slide images
Source: Vet Pathol. 2023 Jul 29;60(6):865–75. doi: 10.1177/03009858231189205 (PMC10583479; doi:10.1177/03009858231189205)
Supplement: sj-pdf-1-vet-10.1177_03009858231189205 – Supplemental material for Automated diagnosis of 7 canine skin tumors using machine learning on H&E-stained whole slide images [file sj-pdf-1-vet-10.1177_03009858231189205.pdf]

Supplemental Materials

Automated diagnosis of seven canine skin tumors using machine learning on H&E-stained whole slide images

Fragoso-Garcia M, Wilm F, Bertram CA, Merz S, Schmidt A, Donovan T, Fuchs-Baumgartinger A, Bartel A, Marzahl C, Diehl L, Puget C, Maier A, Aubreville M, Breininger K, Klopfleisch R

Supplemental Table S1. Total number of annotations created in SlideRunner and the total annotation area for each of the classes in mm^2.

Annotated class	Annotations	Annotation area (mm^2)
Epidermis	3188	2244.57
Dermis	3423	16616.21
Subcutis	2850	7369.88
Trichoblastoma	423	9072.1
SCC	337	3542.28
Melanoma	379	6836.93
Plasmacytoma	377	4750.34
Mast Cell Tumor	161	9330.1
PNST	131	11108.78
Histiocytoma	369	2947.59
Bone	51	216.86
Cartilage	16	32.15
Inflammation/Necrosis	719	2050.16
**Total of annotations**	**12424**	**76118.05**

MCT: mast cell tumor; PNST: peripheral nerve sheath tumor; SCC: squamous cell carcinoma.

Supplemental Table S2. Segmentation confusion matrix.

Prediction						
		Dermis	Epidermis	Subcutis	I/N	Tumor
	Dermis	**0.844**	0.080	0.126	0.150	0.033
	Epidermis	0.009	**0.789**	0.001	0.022	0.001
	Subcutis	0.042	0.005	**0.854**	0.111	0.006
	I/N	0.004	0.008	0.004	**0.456**	0.008
Class	Tumor	0.102	0.117	0.015	0.261	**0.952**
**Segmentation Precision**		**0.844**	**0.789**	**0.854**	**0.456**	**0.952**
**Segmentation Recall**		**0.684**	**0.960**	**0.839**	**0.948**	**0.658**
**F1 Score**		**0.756**	**0.866**	**0.846**	**0.616**	**0.778**

I/N: Inflammation and necrosis.
